# Correction

**DOI:** 10.1080/20008066.2025.2604938

**Published:** 2026-02-27

**Article title:** Symptom retention after successful intensive trauma-focused treatment for post-traumatic stress disorder

**Authors**: Frédérique A. M. Wesseling, Eline M. Voorendonk, Linda Rozendaal and Ad de Jongh

**Journal:** *European Journal of Psychotraumatology*

**DOI***:* https://doi.org/10.1080/20008066.2025.2537546

When it was first published online, a number of errors occurred in **Table 2** and pertain to all odds ratios (ORs) in this table and their interpretation.

The published article reported odds ratios representing: *the probability that if a participant retained a specific symptom at follow-up, they would still meet diagnostic criteria for PTSD after treatment.* However, the intended analysis was: *the probability that a participant would retain a specific PTSD symptom at follow-up given that they no longer met diagnostic criteria for PTSD after treatment**.*** This resulted from a coding error in the analysis script, which inadvertently reversed the conditional probabilities used to compute the odds ratios. As a result, all odds ratios in the originally published Table 2 reflected the **reverse conditional probability**, leading to misinterpretation of symptom persistence. Subsequent reanalysis using the correct conditional probability shows that all calculated odds ratios are below 1, thereby correctly reflecting the *reduced* likelihood of symptom retention after loss of diagnostic status.

The three symptoms most likely to be retained at six-month follow-up when patients no longer met PTSD criteria post-treatment were, in ascending order: C1. Avoidance of thoughts or feelings (OR = 0.03), B1. Intrusive memories (OR = 0.04) and D4. Negative feelings (OR = 0.05). These probabilities were calculated as conditional odds ratios representing the likelihood that a symptom remained present despite the loss of diagnostic status. These changes have also been implemented in the abstract.

In addition to the corrections pertaining to Table 2, the sentence “All odds ratios were smaller than 1, indicating a negative association between having (no) diagnosis and the presence of a symptom.” was added in Section 2.3 to explicitly specify the direction of the conditional probabilities used in the analyses.

The errors detailed above have been corrected in the online version.

